# Molecular characterization of *Myxobolus cuttacki* (Myxozoa, Myxosporea, Bivalvulida) infecting gill lamellae of minor carp *Labeo bata* (Ham.)

**Published:** 2014-12

**Authors:** Sandya Chinna Rajesh, Sayani Banerjee, Avijit Patra, Gadadhar Dash, Thangapalam Jawahar Abraham

**Affiliations:** Department of Aquatic Animal Health, Faculty of Fishery Sciences, West Bengal University of Animal and Fishery Sciences, Kolkata 700094, India

**Keywords:** *Labeo bata*, Myxobolus cuttacki, 18S rRNA, Phylogenetic relationship

## Abstract

As new pathogenic strains are emerging and threatening aquaculture development, myxosporeans (Myxozoa) are receiving much attention in recent years. Myxosporean taxonomy is traditionally based on morphology of the myxospore stage. Molecular data on Indian myxosporeans are rare. In this report, the 18S rRNA gene sequence of Myxobolus cuttacki infecting gill lamellae of minor carp *Labeo bata* (Ham.) and its phylogenetic relationship with other myxobolids are described for the first time. The plasmodia of *M. cuttacki* were 0.5-0.9 mm in size and whitish with a round to oval shape. The mean mature spore size was 16.10×7.05 μm. The 18S rRNA nucleotide sequence with 1703 bp of *M. cuttacki* (accession number KF465682) clustered phylogenetically with other *Myxobolus* spp. infecting cyprinid gills with 78-90% homogeneity. The gill lamellae infecting *M. catmrigalae* (KC933944) and *M. orissae* (KF448527) of Indian major carp *Cirrhinus mrigala* from India, exhibited 86% and 81% homogeneity with *M. cuttacki*, respectively. The infection rate was low to moderate on the gills which can have a negative impact on respiratory and physiological functions and subsequently on fish production.

## INTRODUCTION

Myxosporeans (Phylum: Myxozoa) are microscopic, multicellular, spore-forming parasites of aquatic animals characterized as host, organ and tissue specific organisms [1]. They are identified traditionally based on their myxospore stage morphology, but sometimes, the use of such methods makes the characterization of morphologically similar myxozoans that inhabit taxonomically related host species very difficult [2]. Given the new insights provided by the expanding data set of DNA sequences, myxosporean taxonomy is in a state of flux [3-7]. Over the years, the list of Indian myxosporean species has increased [8-11]. Molecular studies on Indian myxosporeans are rare. Earlier, we reported the molecular characterization of fin-infecting *Thelohanellus caudatus* from carp [12]. In the present work, the molecular characterization of *Myxobolus cuttacki* that infects gill lamellae of minor carp, bata (*Labeo bata* Ham.) is reported together with its phylogenetic relationship.

## MATERIALS AND METHODS

During the routine survey work on parasitic infection of carps, a *Myxobolus* species infecting gill lamellae of minor carp, bata (*Labeo bata*), which was collected from a composite fish culture pond in Garia, Kolkata (Lat. 22°27’59’’N; Long. 88°24’18’’E), West Bengal, India, was characterized by morphometric and molecular techniques. A total of 60 juvenile to sub-adult bata were screened during the survey in March 2013. Myxosporean identification was performed according to Lom and Arthur [13]. Details on spore collection, slide preparation, extrusion of polar filament, detection of iodinophylic vacuoles, staining, permanent mounting, micrometry and molecular characterization are as described in Mondal et al. [12]. Universal eukaryotic primers UEP-F, 5´-ACC TGG TTG ATC CTG CCA G-3´ and UEP-R, 5´-CTT CCG CAG GTT CAC CTA CGG-3´ [14] were used for the amplification of 18S rRNA by Eppendorf Master Cycler Pro S. The PCR amplified product was sequenced at the Genomics Division, Xcelris Labs Ltd., Ahmedabad, India. Following the purification of the amplified PCR product by EXO-SAP treatment, DNA was quantified and subjected to automated DNA sequencing by an ABI 3730xl Genetic Analyzer. BigDye® Terminator v3.1 Cycle sequencing kit (Applied Biosystems, USA) was used for sequencing as per the manufacturers’ instructions. Electrophoresis and data analysis were carried out on the ABI 3730xl Genetic Analyzer. 

Phylogenetic analysis was performed on a selection of 18S rRNA sequences that comprised the new sequence (KF465682) and 25 additional sequences from closely related species available in the NCBI GenBank database using the basic local alignment search tool (BLAST) and other representatives of the Myxobolidae clade (Table 1) as described by Fiala [5]. *Buddenbrockia plumatellae *(AY074915), of the class Malacosporea, was used as an out-group. Data analysis and multiple alignments were performed by ClustalX [15] and MEGA5 [16] softwares, respectively. Genetic distance analyses were conducted using the Kimura 2-parameter model [17]. Included codon positions were 1^st^+2^nd^+3^rd^+Noncoding. All positions containing gaps and missing data were eliminated. The evolutionary history was inferred using the maximum likelihood (ML) method. The bootstrap consensus tree inferred from 1000 replicates was taken to represent the evolutionary history of the analyzed taxa. Branches corresponding to partitions reproduced in <50% bootstrap replicates were collapsed. The percentage of replicate trees in which the associated taxa clustered together in the bootstrap test (1000 replicates) was shown next to the branches [18]. The nucleotide sequence generated in the present study has been deposited in NCBI GenBank database under accession number KF465682.

**Table 1:** Homogeneity of 18S rRNA gene sequences of *Myxobolus cuttacki *(Accession number KF465682) and other Myxobolids and related taxa available in NCBI GenBank

**Myxozoan species**	**NCBI GenBank accession number**	**Site of infection, habitat and host**	**Query coverage (%)**	**DNA sequence homogeneity** **(%) to *Myxobolus cuttacki***
*Myxobolus koi *	FJ841887	G, F, C	85	82
*Myxobolus orissae *	KF448527	G, F, C	85	81
*Myxobolus longisporus *	AY364637	G, F, C	85	82
*Myxobolus ampullicapsulataus *	KC425225	G, F, C	85	81
*Myxobolus wulii *	HQ613412	G&H, F, C	85	82
*Myxobolus bilobus *	DQ008579	G, F, C	85	81
*Myxobolus obesus *	AY325286	G, F, C	65	82
*Myxobolus intimus *	JX390691	G, F, C	65	82
*Myxobolus * *alvarezae*	FJ716096	G, F, C	65	82
*Myxobolus tambroides *	JX028236	G, F, C	65	82
*Myxobolus pesudodispar *	AF380145	M, F, C	85	80
*Myxobolus musculi *	AF 380141	M, F, C	56	80
*Myxobolus cyprini *	AF380140	M, F, C	56	80
*Myxobolus catmirgalae *	KC933944	G, F, C	38	86
*Myxobolus pavlovskii *	HM991164	G, F, C	98	90
*Thelohanellus kitauei *	HQ115585	I&G, F, C	65	88
*Thelohanellus caudatus *	KC865607	Fi, F, C	31	87
*Thelohanellus hovorkai *	DQ231155	A&G, F, C	65	88
*Thelohanellus wuhanesis *	HQ613410	S&G, F, C	96	86
*Myxobolus cerebralis *	AF115254	B, F, Ma, Sa	57	80
*Henneguya ictaluri *	AF195510	G, F, Si	81	78
*Myxdium incurvatum *	DQ377708	Gb, Ma, P	31	86
*Myxidium gadi *	GQ890675	Gb, Ma, Ga	31	86
*Ceratomyxa shasta *	AF001579	Io, F, Sa	26	84
*Buddenbrockia plumatellae *	AY074915	Ma, Bz	30	80

**Note:** G: Gill, M: Muscle, Fi: Fin; B: Brain; H: Hepatopancreas, I: Intestinal wall, Io: Internal organs, A: Abdomen, S: Skin, Gb: Gall bladder, F: Freshwater, Ma: Marine, C: Cypriniformes, Sa: Salmoniformes; Si: Siluriformes; P: Perciformes, Ga: Gadiformes; Bz: Bryozoans

## RESULTS AND DISCUSSION

**Figure 1:**
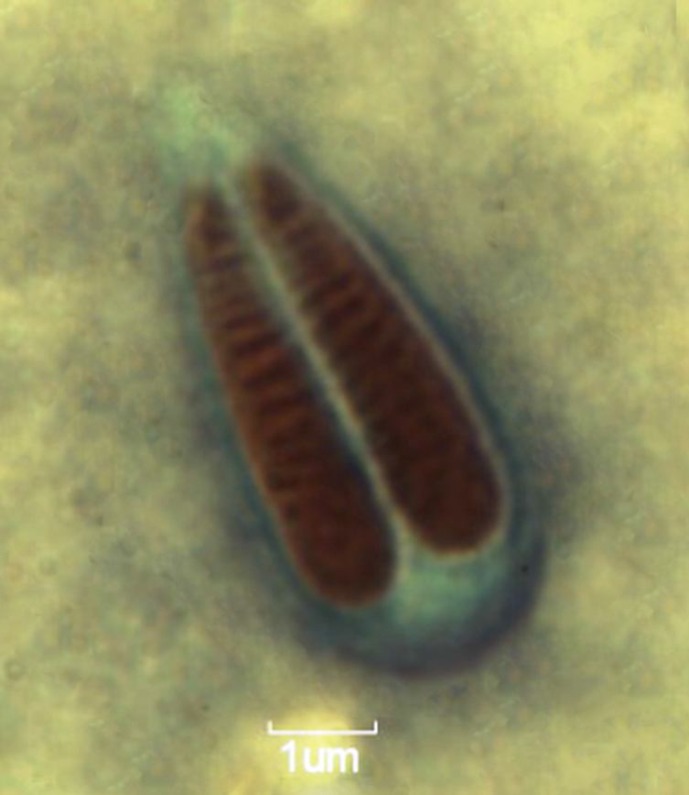
Mature spores of *Myxobolus cuttacki *from fresh wet mount preparation (bar = 1 µm

Of the 60 juvenile to sub-adult bata screened, 25 (41.66%) had low to moderate gill myxosporean infection. The plasmodia were 0.5-0.9 mm in size and whitish round to oval shaped. The mature spore measured 13.40-18.90 (16.10±1.71) × 5.30-8.40 (7.05±1.11) μm. The spores were anteriorly elongated and pointed, and oval to spherical. Shell valves were thick, symmetrical and smooth without any parietal folds. Intercapsular processes were absent*. *Two polar capsules were distinctly equal measuring 6.70-12.30 (9.40±1.56) × 2.00-3.10 (2.50±0.39) μm. The nucleus length was 2.50 μm. Capsules were broadly pyriform with pointed anterior and rounded posterior ends. Polar filaments formed 8–11 coils inside the polar capsules and were coiled perpendicularly to the axis of the capsules. The spore length (LS) to spore breadth (BS) ratio was 1:0.43, while the polar capsules length to breadth (LPC and BPC) ratio was 1:0.26. The capsules opened independently. Both polar capsules were situated parallel to each other in the spore cavity (Fig. 1). 

**Figure 2:**
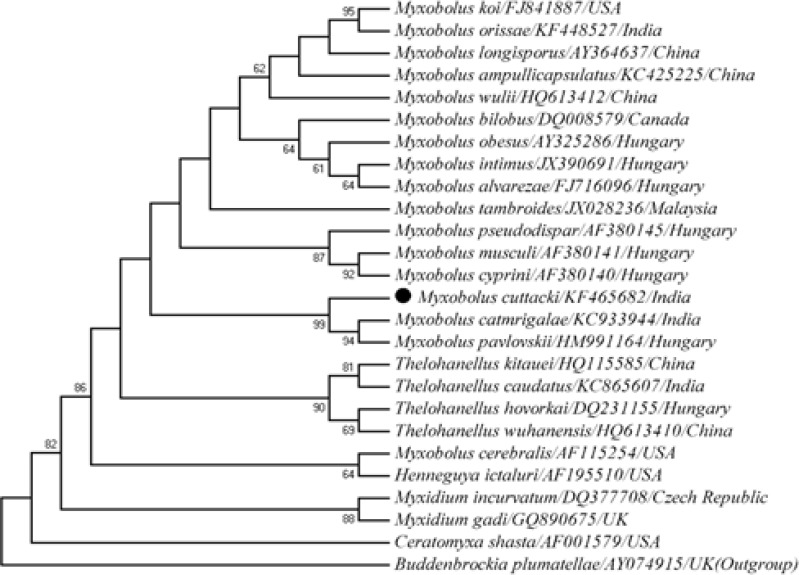
Phylogenetic tree generated by maximum likelihood of the 18S rRNA gene sequences of *Myxobolus cuttacki *(NCBI Accession number KF465682) and other cyprinids gill, fin and muscle infecting* Myxobolus *spp., and related taxa. Bootstrap confidence values are shown at nodes (1000 replications).

The universal eukaryotic primer sets UEP-F and UEP-R successfully amplified approximately 2048 bp fragments of the 18S rRNA gene from *M. cuttacki*. Phylogenetic cluster was established on the basis of consensus sequence, which was 1703 bp in length. The DNA sequence of *M. cuttacki *clustered phylogenetically with other *Myxobolus *spp. infecting the cyprinid gills (Fig. 2) with 78-90% homogeneity (Table 1). The out-group *B. plumatellae *(AY074915), of the class Malacosporea, was phylogenetically clustered distinctly as a separate lineage with Myxosporea (Fig. 2). Evolutionary pair-wise distances among *M. cuttacki* and other analyzed species, measured by Kimura-2-Parameter algorithm, were in the range of 0.00 - 3.00 (Table 2).

Although morphological data on many Indian *Myxobolus *spp. have been studied [8-11], molecular data have not been fully explored yet. Several gill-infecting *Myxobolus *spp. with equal polar capsules are known from Indian cyprinids [9]. The morphometry and spore index values of the present species were, more or less, in conformity with the original descriptions of *M. cuttacki* (LS:BS =1:0.37; LPC:BPC = 1:0.32), a species described by Haldar et al. [19], isolated from *Cyprinus carpio *in Orissa, India. However, they differed slightly in spore size, possibly due to the use of image analyzing software. Nevertheless, the limits of natural variations typical of populations or species, which are influenced by several factors such as differences in location, ecology, ecological condition and age of the fish were not exceeded [20]. Comparison of the morphometric data of *M. cuttacki* of the present study with the representatives of *Myxobolus* spp. infecting the gills of cyprinids [9] revealed further differences in its morphometric characteristics from others. These observations, thus, confirmed that the *Myxobolus* species found on the gills of cultured bata was *M. cuttacki* in its morphology, host (carp) specificity and tissue (gill) tropism. 

**Table 2:** Estimates of evolutionary divergence between the sequences of Myxosporea and Malacosporea available in NCBI GenBank database

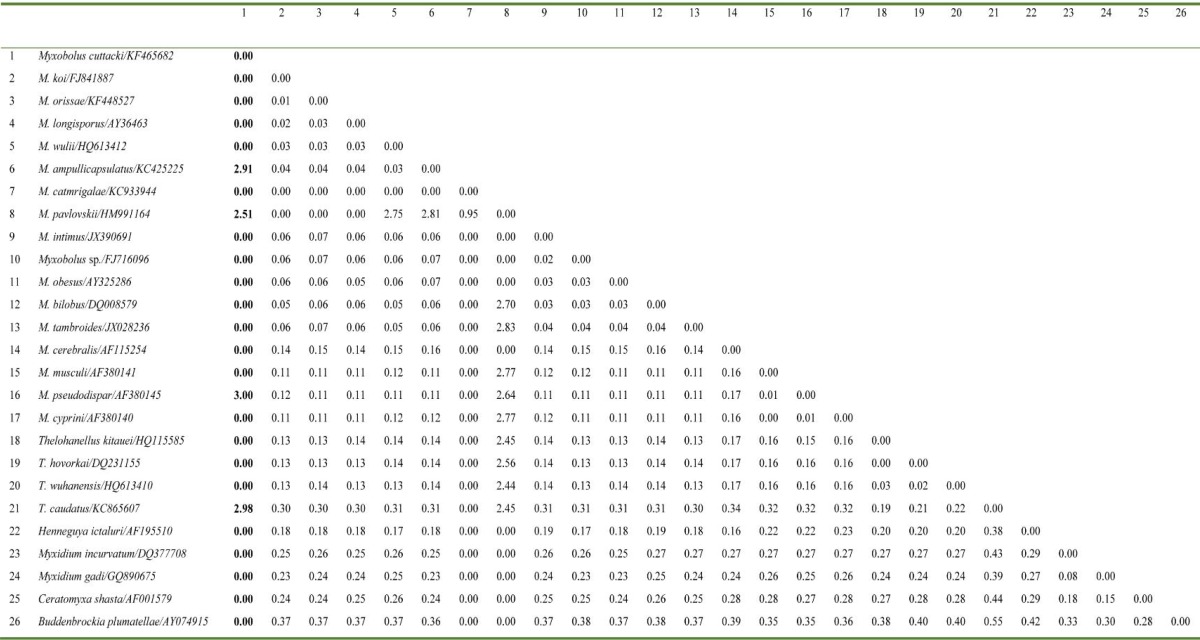

Earlier, we characterized *M. catmrigalae *(KC933944) and* M. orissae *(KF448527), both infecting gill lamellae of carp, *Cirrhinus mrigala. *In this study, the molecular characterization of yet another Indian myxosporean,* M. cuttacki* that infects carp gill lamellae is described. *Myxobolus *representatives cluster according to their site specificity rather than their spore morphology. As per Eszterbauer [4], this specificity is an important factor in myxozoan speciation. The phylogenetic tree illustrated the taxonomic placement of a series of myxobolids based on the analysis of a small subunit 18S rRNA, and was very similar to and defined the topologies resembling those generated by Fiala [5]. The evolutionary tree of this study demonstrated that tissue (gill) tropism may play an important role in genetic relationships among myxozoan species. The phylogenetic tree placed *M. cuttacki* within the freshwater clade. It formed a dichotomy with gill-infecting *M. catmrigalae* (KC933944), its closest relative. Our previously characterized *M. catmrigalae* (KC933944) and *M. orissae* (KF448527) exhibited 86% and 81% homogeneity with *M. cuttacki, *respectively. The maximum homogeneity (90%) was shown by *M.* *pavlovskii* (HM991164) infecting gill lamellae of silver carp, *Hypophthalmichthys molitrix* from Hungary*.* Furthermore, freshwater and marine clades (*Myxidium incurvatum* (DQ377708) and *Myxidium gadi* (GQ890675)) were distinctly separated within the lineage Myxosporea. Other gill-infecting *Myxobolus *spp. were observed to be distantly related to *M. cuttacki. *All skeletal muscle infecting myxosporeans, viz., *M. cyprini *(AF380140), *M. musculi *(AF380141) and *M. pseudodispar* (AF380145) were distinctly different from gill-infecting *Myxobolus *spp., and phylogenetically clustered together as a separate subclade. Other representatives of the Myxobolidae clade such as *Ceratomyxa, Henneguya, Myxidium* and *Thelohanellus* were also distinctly different from gill-infecting *Myxobolus *spp. and clustered separately. The observed evolutionary pair-wise distances among *M. cuttacki* and other analyzed species ranging from zero, with the majority of the myxobilids including *M. catmrigalae*, to 3.00, with *M. pseudodispar, *possibly indicate high genetic diversity among myxosporeans.

The need for more accurate diagnosis of myxosporeans has increased recently due to insufficient morphological and molecular characterizations of many *Myxobolus* species [3]. Myxosporeans are best known for the diseases they cause in commercially important fish hosts [7, 9]. With the huge expansion of freshwater aquaculture in India, several myxosporeans have been recognized, or elevated in status, as important pathogens [9]. Since the gills of bata had low to moderate infections with a parasitic frequency index of 41.66%, negative effects on the respiratory and physiological functions can be generally assumed. Although no mortality was noticed in the present study, the results further suggest that cultured bata might be under stress due to unfavourable growth conditions. 

This communication is the first to report on molecular and phylogenetic characterizations of *M. cuttacki*. Similar works on Indian myxosporeans are currently in progress, which could provide baseline data for future research on molecular taxonomy, phylogenetic relationships, genetic diversity, molecular pathogenesis, etc.

## Notes

**Acknowledgments: **This work was supported by the Indian Council of Agricultural Research, Government of India, New Delhi, under the Niche Area of Excellence program. The authors thank the Vice-Chancellor of the West Bengal University of Animal and Fishery Sciences, Kolkata, for providing necessary infrastructure facilities to carry out the work.
